# Fungal Enolase, β-Tubulin, and Chitin Are Detected in Brain Tissue from Alzheimer’s Disease Patients

**DOI:** 10.3389/fmicb.2016.01772

**Published:** 2016-11-07

**Authors:** Diana Pisa, Ruth Alonso, Alberto Rábano, Michael N. Horst, Luis Carrasco

**Affiliations:** ^1^Centro de Biología Molecular “Severo Ochoa,” Universidad Autónoma de MadridMadrid, Spain; ^2^Department of Neuropathology and Tissue Bank, Unidad de Investigación Proyecto Alzheimer, Fundación CIEN, Instituto de Salud Carlos IIIMadrid, Spain; ^3^Division of Basic Medical Sciences, Mercer University School of Medicine, MaconGA, USA

**Keywords:** neurodegenerative disease, fungal infection, Alzheimer’s disease, fungal proteins, chitin

## Abstract

Recent findings provide evidence that fungal structures can be detected in brain tissue from Alzheimer’s disease (AD) patients using rabbit polyclonal antibodies raised against whole fungal cells. In the present work, we have developed and tested specific antibodies that recognize the fungal proteins, enolase and β-tubulin, and an antibody that recognizes the fungal polysaccharide chitin. Consistent with our previous studies, a number of rounded yeast-like and hyphal structures were detected using these antibodies in brain sections from AD patients. Some of these structures were intracellular and, strikingly, some were found to be located inside nuclei from neurons, whereas other fungal structures were detected extracellularly. Corporya amylacea from AD patients also contained enolase and β-tubulin as revealed by these selective antibodies, but were devoid of fungal chitin. Importantly, brain sections from control subjects were usually negative for staining with the three antibodies. However, a few fungal structures can be observed in some control individuals. Collectively, these findings indicate the presence of two fungal proteins, enolase and β-tubulin, and the polysaccharide chitin, in CNS tissue from AD patients. These findings are consistent with our hypothesis that AD is caused by disseminated fungal infection.

## Introduction

Despite intensive research effort, the precise etiology of Alzheimer’s disease (AD) remains unknown. AD will affect over 60 million people worldwide by 2030 unless a remedy is found. Substantial progress has been made in understanding the basic mechanisms of AD and the prevailing hypothesis for AD pathogenesis is the amyloid cascade, in which secretion of amyloid β peptide (Aβ) gives rise to the extracellular deposition of amyloid plaques that in turn induce the aggregation of phosphorylated tau protein (O’Brien and Wong, 2011; Hampel et al., 2015). Formation of intracellular tangles of phosphorylated tau leads to neuronal death and progressive neurodegeneration (Revett et al., 2013). Although broadly accepted as a pathogenic mechanism in AD, a number of studies have provided evidence for microbial infection as the main etiological factor in AD (Harris and Harris, 2015; Olsen and Singhrao, 2015). Among them, viruses, herpesviruses and particularly herpes simplex virus type 1 (HSV-1) have been the focus of investigation (Itzhaki, 2014; Piacentini et al., 2014; Harris and Harris, 2015). Along this line, HSV-1 DNA is found in AD patients (Wozniak et al., 2009), but whether this infection causes AD is controversial since the vast majority of the human population bears this viral DNA in brain tissue (Marques et al., 2001; Hemling et al., 2003; Wozniak et al., 2005). Another possibility considered by some is that bacteria such as *Chlamydophila pneumoniae* or spirochetes are the etiological agents of AD (Balin et al., 2008; Miklossy, 2011). This proposition is based on the finding that *C. pneumoniae* structures and DNA are present in AD brain tissue (Balin et al., 1998; Gerard et al., 2006); however, this has been questioned by other researchers (Gieffers et al., 2000; Ring and Lyons, 2000). The finding that Aβ peptide exhibits antibacterial and anti-fungal activity point to the possibility that amyloid plaque formation is a response to microbial infection (Soscia et al., 2010). Indeed, Aβ peptide expression protects against fungal and bacterial infections in expermental animal models (Kumar et al., 2016). A model in which Aβ has a protective-damaging action has been suggested.

The consideration that fungal infection is responsible for the pathology observed in several neurodegenerative disorders, including AD, has received scant attention. We previously demonstrated that fungal proteins and DNA can be detected in blood serum and cerebrospinal fluid (CSF) from AD patients (Alonso et al., 2014a, 2015a). Additionally, proteomic analyses revealed the presence of fungal proteins such as tubulin in brain tissue, and fungal DNA was also detected by PCR analyses (Alonso et al., 2014b). Through this analysis, several fungal species were detected, suggesting that mixed disseminated mycoses exists in the central nervous system (CNS) of AD patients. Moreover, a number of fungal structures can be directly visualized both inside and outside of neurons by immunohistochemistry with rabbit polyclonal antibodies (Pisa et al., 2015a,b). Thus, yeast-shaped cells and hyphae are evident in brain tissue from patients, but not in control subjects. The antibodies employed in these studies were raised against whole fungal cells and, consequently, several different proteins were visualized. These non-specific antibodies crossreact with other fungal species, making them broad-spectrum. This lack of specificity may represent a starting point to identify different structures from a variety of fungal species. The use of specific antibodies that recognize individual fungal components would be a next step in evaluating possible fungal infection. Accordingly, in the present study we have developed and tested three antibodies to detect fungi in brain samples. One antibody has been raised against the fungal polysaccharide chitin and two antibodies have been developed against fungal proteins: one raised against purified enolase from *Candida famata* and the other raised against a β-tubulin peptide specific for fungi. Using these three novel antibodies, we provide further support for presence of fungal structures in brain tissue from AD patients, but not in control subjects.

## Materials and Methods

### Description of Control Subjects and Patients

We analyzed samples from patients diagnosed with AD and control individuals without neurological disease. The age and gender of the subjects are listed in **Supplementary Table S1**. All samples were supplied by a brain bank (Banco de Tejidos CIEN, Madrid) and were analyzed anonymously. The transfer of samples was carried out according to national regulations concerning research on human biological samples. The Ethics Committee of the Universidad Autónoma de Madrid approved the study. In all cases, written informed consent was obtained.

### Development of Anti-fungal Antibodies

Anti-chitin antibodies were generated as previously described (Walker et al., 1990, 1991). Briefly, rabbits (female New Zealand) were immunized with reacetylated chitosan suspended in phospate-buffered saline (PBS; 20 mM sodium phosphate buffer, pH 7.4, 0.15 M NaCl). After weekly subcutaneous injection (0.8 ml of PBS containing 0.1–0.6 mg reacetylated chitosan) for 4 weeks, animals were ear bled using a vacuum cuff. The antiserum was obtained and purified by affinity chromatography on protein A-agarose. Bound antiserum was eluted with 0.2 M glycine buffer, pH 2.9, neutralized, and dialyzed. To obtain the fraction that bound di-N-acetylchitobiose, the antiserum was further purified by affinity chromatography on ovalbumin-agarose. The unbound material, which had no binding activity toward ovalbumin (undetectable by western blotting), was stored at -20°C until needed. These antibodies were obtained at Mercer University (USA) before IACUC was instituted. Nevertheless, we followed all USDA-approved animal care regulations regarding immunization and subsequent ear-bleeding at that time.

Antibodies against *C. famata* enolase were obtained by injection of a purified fusion protein (maltose-binding protein plus enolase). The enolase gene was obtained by PCR amplification using the primers CGCCGCGGATCCATGGCCGTCACTAAGTTATT and CGCCGCGTCGACTTATAATTGAGAAGCAGCGT. The amplifed fragment was cloned into the pMAL vector (New England Biolabs, Ipswich, MA, USA). After expression, the fusion protein was purified on maltose columns and eluted with 0.5 ml PBS. Rabbits were injected every 3 weeks with 1 mg of fusion protein suspended in Freund’s adjuvant. A peptide corresponding to a region of fungal β-tubulin (DVVRREAEGCDS) was purchased from PolyPeptide Group (Strasbourg, France) and conjugated to keyhole limpet hemocyanin (KLH). This peptide sequence is conserved in several fungal species and is absent in human β-tubulin. Rabbits and rats were inoculated with 0.60 mg of β-tubulin peptide-KLH previously mixed with an equal volume of Freund’s adjuvant every 2 weeks, up to three times in total. The antibody titer and specificity of the antiserum were tested by immunohistochemistry. The protocols employed were approved by the ethics committee of Centro de Biologia Molecular “Severo Ochoa” (identification number: ES280790000180). Animal welfare and methods for sacrifice dictated by the European Union have been followed. Animals were injected with sodium pentothal as the anesthetic before bleeding.

### Immunohistochemistry Analysis

CNS tissue was embedded in paraffin following standard techniques and cut into 5 μm sections using a microtome (Microm HM355s; Microm, Walldorf, Germany). For immunohistochemical analysis, paraffin was removed and sections were rehydrated and boiled for 2 min in 10 mM citrate buffer and then incubated for 10 min with 50 mM ammonium chloride. Tissue sections were then incubated for 10 min with PBS/Triton X-100 (0.1%) followed by 20 min with 2% bovine serum albumin in PBS. Sections were incubated overnight at 4°C with mouse monoclonal antibodies against human α-tubulin (Sigma), human phospho-PHF-tau, clone AT100 (Thermo Scientific), human neurofilament protein, clone 2F11 (Dako), or rabbit polyclonal antibodies raised against enolase, β-tubulin, or chitin, all at 1:50 dilution. Thereafter, sections were washed with PBS and further incubated for 1 h at 37°C with donkey anti-mouse IgG secondary antibody conjugated to Alexa 555 (Invitrogen) at 1:500 dilution for α-tubulin, tau and neurofilament, and donkey anti-rabbit IgG secondary antibody conjugated to Alexa 488 (Invitrogen) at 1:500 dilution for anti-fungal antibodies. To visualize nuclei, sections were stained with 4,6-diamino-2-phenylindole (DAPI; Merck) and treated with autofluorescence eliminator reagent (Merck). The use of this reagent is important to avoid autofluorescence since lipofuscin is present in the aging brain. All images were collected and analyzed on a Zeiss LSM710 confocal laser scanning microscope equipped with the upright microscope stand AxioImager.M2 (Zeiss), running ZEN 2010 software. The spectral system employed was Quasar + 2 PMTs. Images were deconvoluted using Huygens software (4.2.2 p0; Scientific Volume Imaging) and visualized and processed with Fiji/ImageJ software (NIH, Bethesda, MD, USA). Stacks of 3D images were collected with the high-speed, high-resolution A1R+ confocal microscope (Nikon) combined with an inverted microscope, running NIS Elements 4.40 software.

### Western Blotting Assay

Fungal proteins were precipitated with 10% trichloroacetic acid. HeLa cells were grown in Dulbecco’s modified Eagle medium supplemented with 10% fetal calf serum. These cells were collected in sample buffer (0.37 M Tris–HCl, pH 6.8, 0.1 M DTT, 2% SDS, 17% glycerol, and 0.024% bromophenol blue) and boiled for 5 min. Fungal and HeLa cell proteins were fractionated by SDS-PAGE (15% polyacrylamide), transferred to nitrocellulose membranes by wet immunotransfer and processed for western blotting after blocking the membranes with 1% bovine serum albumin. Incubation with specific rabbit antibodies for peptide β-tubulin-KLH and enolase at a 1:200 dilution and specific mouse antibody for human α-tubulin at a 1:500 dilution was performed for 1 h. Donkey anti-rabbit and sheep anti-mouse IgG horseradish peroxidase-conjugated antibodies (Amersham Biosciences) and the ECL kit (Amersham) were used to detect bound antibodies.

## Results

### Characterization of Anti-fungal Antibodies

Our previous findings strongly indicated the presence of fungal structures in brain tissue from AD patients as revealed by positive staining with antibodies raised against whole fungal cells (Pisa et al., 2015a,b). Although the antibodies were specific and did not recognize human antigens, the number of proteins and other cellular components immunoreacting with the polyclonal antibodies were unknown. We therefore generated three antibodies against individual fungal components to further evaluate the existence of these specific proteins/polysaccharides in brain tissue. Anti-chitin antibodies have been successfully tested against a number of fungal pathogens (Walker et al., 1990, 1991) and selectively recognize chitin, which is not present in human tissues. Additionally, we raised an antibody against the glycolytic enzyme enolase cloned from *C. famata*, and against a peptide specific for fungal β-tubulin. The three rabbit polyclonal antibodies were first tested using immunofluorescence microscopy on different *Candida* species. Anti-chitin, immunoreacted strongly with *C. parapsilosis* and *C. tropicalis*, while its reactivity with *C. albicans. C. krusei*, and *C. glabrata* was weaker, suggesting that the chitin present in the latter yeast species is differentially recognized by the antibody perhaps due to differences in the structure or in the amount of chitin present in the cell wall (**Supplementary Figure S1A**). Additionally, the anti-enolase antibody immunoreacted strongly with *C. krusei* and *C. glabrata*, whereas the anti-β-tubulin antibody immunoreacted strongly with *C. albicans. C. tropicalis*, and *C. krusei*. Rat polyclonal antibodies raised against the β-tubulin peptide also immunoreacted with the different *Candida* spp. (**Supplementary Figure S1A**). Thus, all three rabbit antibodies and a rat antibody immunoreacted with *Candida* spp. to different degrees. Western blotting analyses showed that anti-enolase and anti-β-tubulin antibodies recognized the corresponding protein bands in *C. famata* but not in HeLa lysates (**Supplementary Figure S1B**). Conversely, a commercial anti-human tubulin antibody detected tubulin in HeLa lysates but not in *C. famata*. Further assessment of the selectivity of the three fungal antibodies was performed by testing their reactivity against human HeLa cells by immunocytochemistry. As expected, none of the three antibodies immunoreacted with components of HeLa cells (**Supplementary Figure S1C**).

### Detection of Chitin in Brain Tissue from AD Patients

**FIGURE 1:**
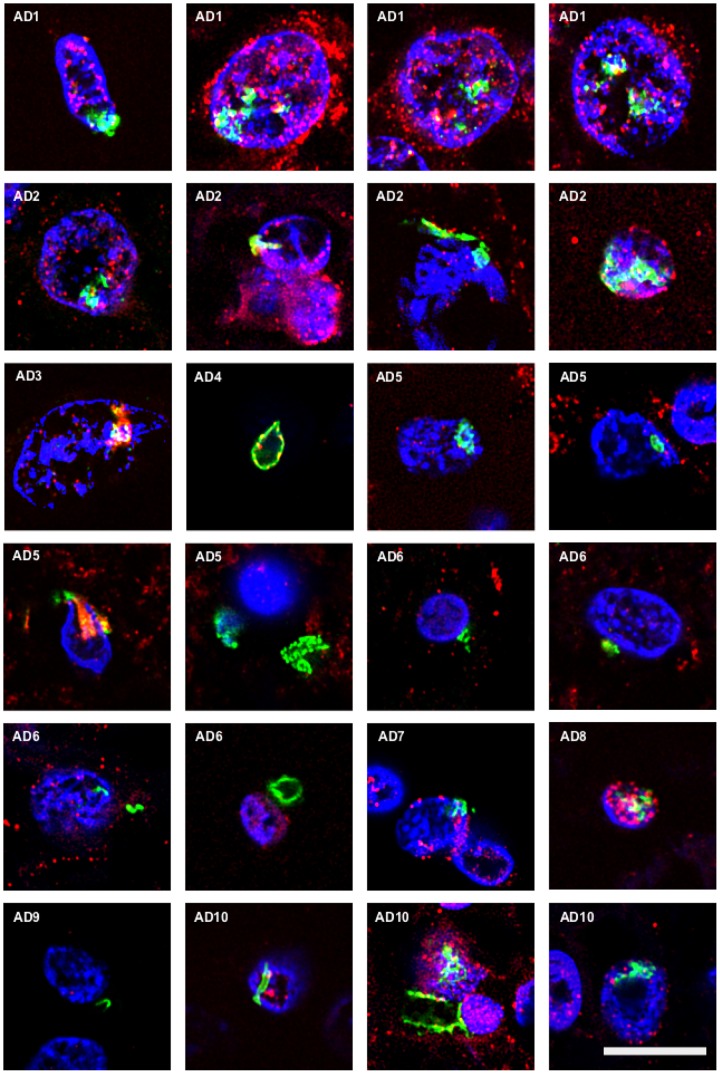
**Identification of fungal chitin in brain sections of AD patients by immunohistochemistry.** Entorhinal cortex (ERH) sections (5 μm) from 10 different AD patients were immunostained for 1 h with rabbit polyclonal anti-chitin antibodies (green) and mouse monoclonal anti-human α-tubulin antibodies (red). DAPI staining of nuclei appears in blue. Double immunostaining and confocal microscopy were carried out as indicated in the section “MATERIALS AND METHODS.” Scale bar: 10 μm.

Chitin is a polysaccharide of N-acetylglucosamine that is an integral part of the fungal cell wall. Chitin-like bodies have been observed in AD brains stained with the flurochrome dye calcofluor (Castellani et al., 2005). Moreover, levels of chitinase (chitotriosidase), a human enzyme synthesized by macrophages, are very high in serum and CSF from AD patients (Choi et al., 2011; Watabe-Rudolph et al., 2012; Rosen et al., 2014; Melah et al., 2015). We therefore tested for the first time specific anti-chitin antibodies to survey potential fungal structures in AD brains. Tissue sections from the entorhinal cortex of 10 AD patients were double stained with an anti-chitin antibody (1:50 dilution, shown in green) and a mouse monoclonal anti-human α-tubulin antibody, which was used to identify microtuble structures (shown in red). Cell nuclei were visualized by DAPI staining (shown in blue; **Figure 1**). A number of structures were stained with the anti-chitin antibody (**Figure 1**); some of which were in the form of punctate bodies or yeast-like structures. On several occasions, the stained structures were located inside the nucleus, while in other sections they were detected around the nucleus or were extracellular. Hyphal structures could also be readily detected with the anti-chitin antibody (**Figure 1**, AD10). In summary, different structures that immunoreacted with the anti-chitin antibody were detected in all 10 AD patients examined. Since the morphology of these structures is similar to fungal cells and hyphae, and they most probably contain chitin, it seems plausible to suspect the existence of mycoses in the entorhinal cortex of the patients.

### Fungal Enolase and β-tubulin Are Observed in AD Brain Tissue

**FIGURE 2:**
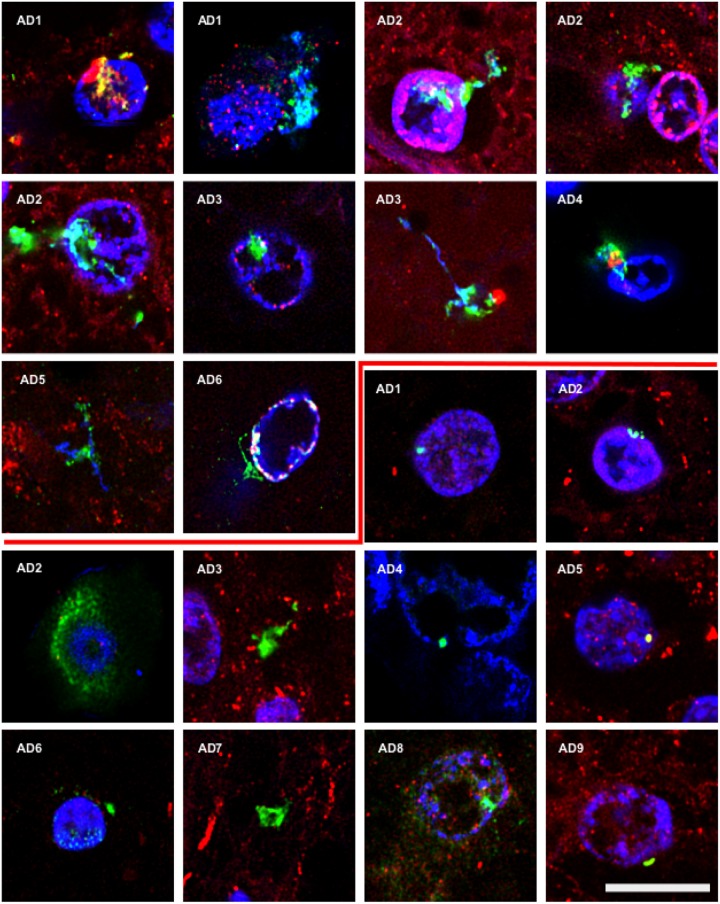
**Detection of fungal enolase and β-tubulin in brain sections from AD patients using specific antibodies.** Entorhinal cortex (ERH) sections (5 μm) from nine different AD patients. Upper panels: samples were immunostained with rabbit polyclonal anti-enolase antibodies (green) and mouse monoclonal anti-human α-tubulin antibodies (red). Lower panels: samples were immunostained with rabbit polyclonal fungal anti-tubulin antibodies (green) and mouse monoclonal anti-human neurofilament antibodies (red). DAPI staining of nuclei appears in blue. Double immunostaining and confocal microscopy were carried out as indicated in the section “MATERIALS AND METHODS.” Scale bar: 10 μm.

To further assess the presence of specific fungal components in AD brain tissue, we employed rabbit polyclonal anti-enolase and fungal anti-β-tubulin antibodies. First, tissue sections of the entorhinal cortex were double stained with an anti-enolase (green) and a human anti-α-tubulin antibody (red; **Figure 2**, Upper panels). Entorhinal sections were also double stained with anti-β-tubulin (green) and a mouse monoclonal anti-neurofilament antibody (red; **Figure 2**, Lower panels). In all cases, nuclei were visualized by DAPI staining (blue). Fungal enolase was clearly detected in brain tissue from AD patients. The immunostained structures were similar to those described with anti-chitin antibodies but the staining was more robust, perhaps due to the better reactivity of the antibody against a protein rather than a polysaccharide. Of particular interest was the finding of intranuclear fungal structures (**Figure 2**, panel AD6). Immunostaining with the anti-β-tubulin antibody highlighted the presence of a number of punctate bodies in the cytoplasm of a neuron (**Figure 2**, Lower panel AD2). The immunostained punctate bodies are reminiscent of those observed after yeast infection of cultured cells or mice (Pacheco et al., 2007; Pisa et al., 2015a). In addition, several rounded fungal cells were clearly visible in neuronal nuclei (**Figure 2**, Lower panels). As controls for the different antibodies employed in this work, immunohistochemistry analyses of AD sections were carried out without primary antibodies. Certainly, in presence of anti-enolase antibodies fungal structures are detected, whereas when this primary antibody is omitted the green structures are not observed (**Supplementary Figure S2**). Thus, when the secondary antibody against rabbit polyclonal antibodies is employed, no fungal structures are revealed. On the other hand, omission of the secondary antibody that recognizes mouse monoclonal antibodies does not visualize human α-tubulin.

To validate these results, we carried out double immunostaining using rabbit polyclonal anti-chitin antibodies and rat polyclonal fungal anti-β-tubulin antibodies. Curiously, whereas some putative fungal structures were recognized by both antibodies, other structures were immunostained with either one or the other of these antibodies (**Supplementary Figure S3**). Moreover, when the same structure was recognized by both antibodies, the pattern of staining observed with each antibody could differ (see Lower panels in **Supplementary Figure S3**).

**FIGURE 3:**
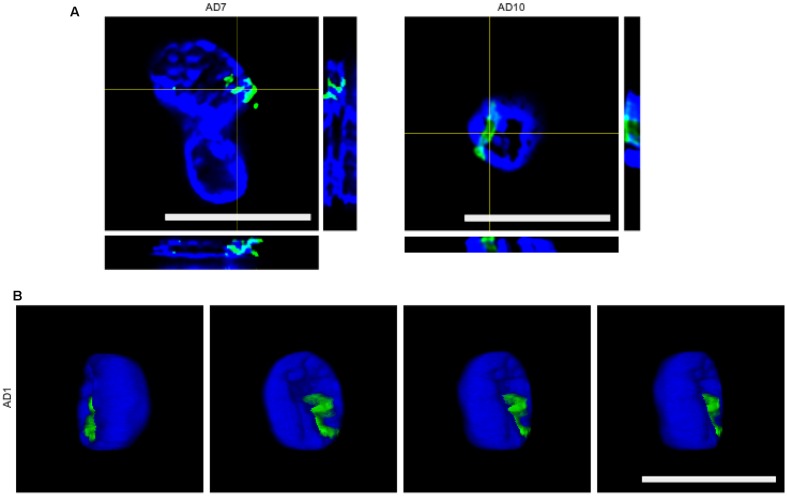
**Orthogonal projections and 3D images.** **(A)** Orthogonal projection of entorhinal cortex of patients AD7 and AD10. Images were acquired with a LSM710 confocal microscope. **(B)** Different stacks of a 3D image of the entorhinal cortex of patient AD1. Images were acquired with a Nikon A1R+ confocal microscope. Rabbit polyclonal anti-chitin antibody staining is shown in green and DAPI staining is shown in blue for all panels. Scale bars: 10 μm.

To further determine whether some of the putative fungal structures were intranuclear, we carried out orthogonal projection analysis. **Figure 3** shows that the anti-chitin antibody immunoreacted with intranuclear material from patients AD7 and AD10 and an intranuclear fungal hypha is clearly visible (**Figure 3A**). Remarkably, 3D analysis of one neuronal nucleus demonstrated the presence of one yeast cell inside the nucleus. Since intracellular infection by fungal cells requires that they be alive, we conclude that infection of the neurons occured when the patient was alive, and was not the consequence of post-mortem contamination.

### Different Regions of the AD Brain Contain Fungal Chitin, Enolase, and β-tubulin

**FIGURE 4:**
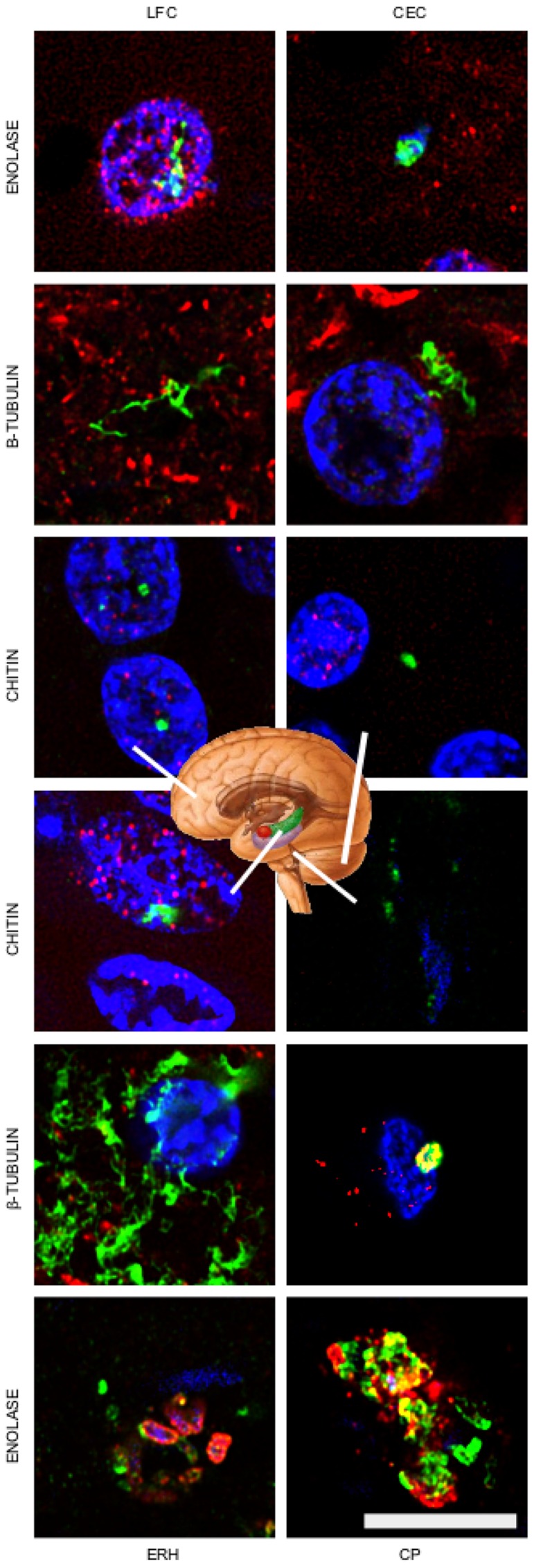
**Detection of fungal enolase, β-tubulin, and chitin in different CNS regions of patient AD11.** Immunohistochemistry analysis of CNS sections from patient AD11 were performed by confocal microscopy as detailed in the section of “MATERIALS AND METHODS.” LFC, lateral frontal cortex; CEC, cerebellar cortex; ERH, entorhinal cortex/hippocampus; CP, choroid plexus. Anti-enolase, anti-tubulin, and anti-chitin antibodies are shown in green. Double staining with mouse monoclonal anti-human α-tubulin inmunostaining for the anti-enolase antibody sample, human anti-neurofilament immunostaining for fungal anti-tubulin antibody sample, and anti-TauT100 staining for human anti-chitin antibody sample are shown in red. DAPI staining appears in blue. The different CNS sections are indicated in the panels. Scale bar: 10 μm.

Having shown that fungal bodies could be detected in the entorhinal cortex of several patients, we next sought to evaluate different CNS regions. To this end, sections from the lateral frontal cortex (LFC), cerebellar cortex (CEC), entorhinal cortex/hippocampus (ERH) and choroid plexus (CP) of one AD patient were immunostained with the three anti-fungal antibodies described above (**Figure 4**, shown in green). For double immunostaining studies, we used monoclonal anti-human phosphorylated tau with anti-chitin, mouse monoclonal anti-human α-tubulin with anti-enolase, and monoclonal neurofilament antibody with anti-β-tubulin (shown in red). Results showed that a number of fungal structures were detected in the four different regions, pointing to the idea that fungal infection is disseminated in different brain regions (**Figure 4**). It is possible that the fungal species and/or the structures recognized by each antibody could be different. Once again, the morphology of the structures immunostained with the anti-fungal antibodies was similar to those described in the entorhinal cortex. Accordingly, intranuclear yeast-shaped cells were found in the LFC with anti-chitin antibodies. Also, hyphal structures were detected in the LFC, CEC and ERH regions with the anti-β-tubulin antibody. In CP, several yeast cells of about 1–2 μm are evidenced inside a blood vessel using the anti-enolase antibody. Collectively, these results lead us to conclude that specific fungal macromolecules such as chitin and proteins are detected in fungal structures widespread in different brain regions from the AD patient analyzed.

### Fungal Components in Corpora Amylacea

**FIGURE 5:**
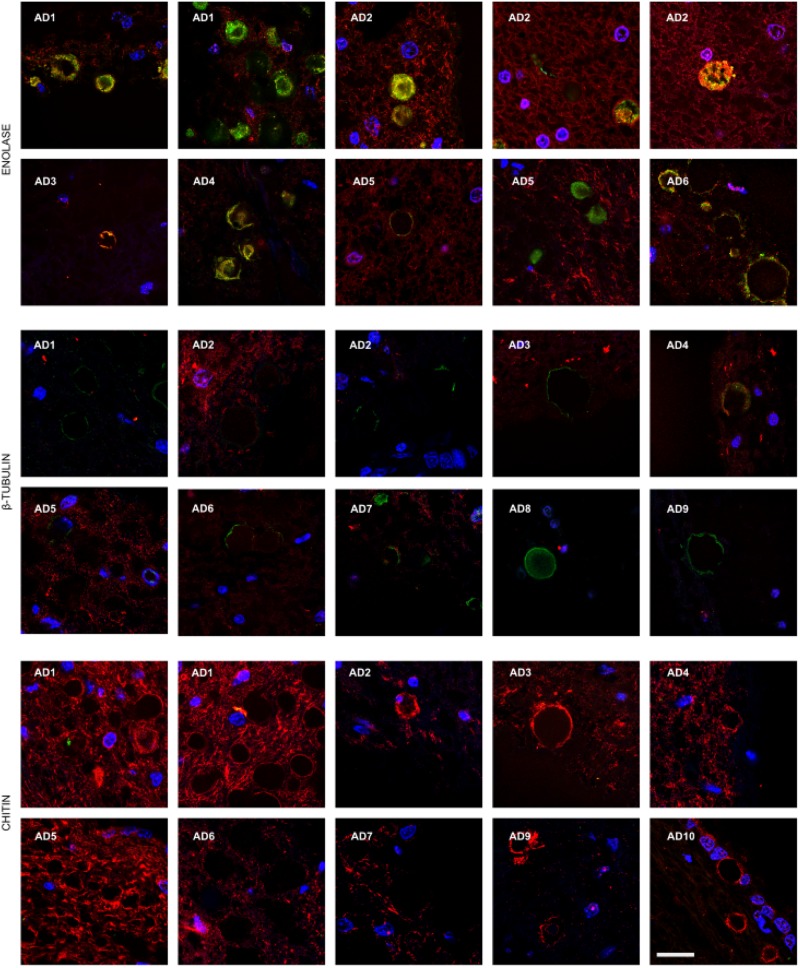
**Presence of fungal enolase and *β*-tubulin in corpora amylacea.** Entorhinal cortex (ERH) sections from 10 AD patients were incubated with rabbit polyclonal antibodies against fungal enolase, β-tubulin, and chitin as described in the section of “MATERIALS AND METHODS” (green), and mouse monoclonal antibodies against human α-tubulin and neurofilaments used as described in **Figures 1** and **2**, respectively, is shown in red. DAPI appears in blue. Scale bar: 20 μm.

Corpora amylacea (CA) are small rounded bodies of 10–50 μm that are very abundant in the CNS of patients with neurodegenerative diseases, including AD (Rohn, 2015; Pisa et al., 2016). The composition of CA has been analyzed in some detail. They mainly contain polyglucans and only a small percentage (4%) corresponds to proteins (Robitaille et al., 1980; Nishimura et al., 2000). The precise origin of CA remains enigmatic, but it is thought that they accumulate in elderly people and their formation occurs over long periods (Sfanos et al., 2009). We recently found that fungal proteins can be detected in CA with polyclonal antibodies raised against whole fungal cells (Pisa et al., 2016). Thus, it was of interest to examine whether specific fungal components could be detected in CA using the new antibodies described here. Thus, CA were localized from entorhinal cortex brain sections and were immunostained as described (**Figures 1** and **2**). Of note, fungal enolase could be clearly detected in the vast majority of CA, which showed a high immunoreactivity with the antibody (**Figure 5**, Upper panels). By contrast, CA showed low reactivity with the anti-β-tubulin antibody, and not all CA were immunolabeled (**Figure 5**, Middle panels). Curiously, anti-chitin reactivity was not detected in any of the CA, suggesting that chitin is not incorporated into these bodies (**Figure 5**, Lower panels). Therefore, we conclude that some specific fungal proteins can be detected in CA, and enolase is a particularly good marker to use for detection. This may be due to the fact that enolase is both a secreted and cytoplasmic protein, whereas β-tubulin is cytoplasmic and forms part of the fungal cytoskeleton. The finding that fungal chitin is not found in CA may be due to the fact that CA are mainly composed of polyglucans.

### Analysis of Anti-fungal Antibody Reactivity in Brain Tissue from Control Subjects

**FIGURE 6:**
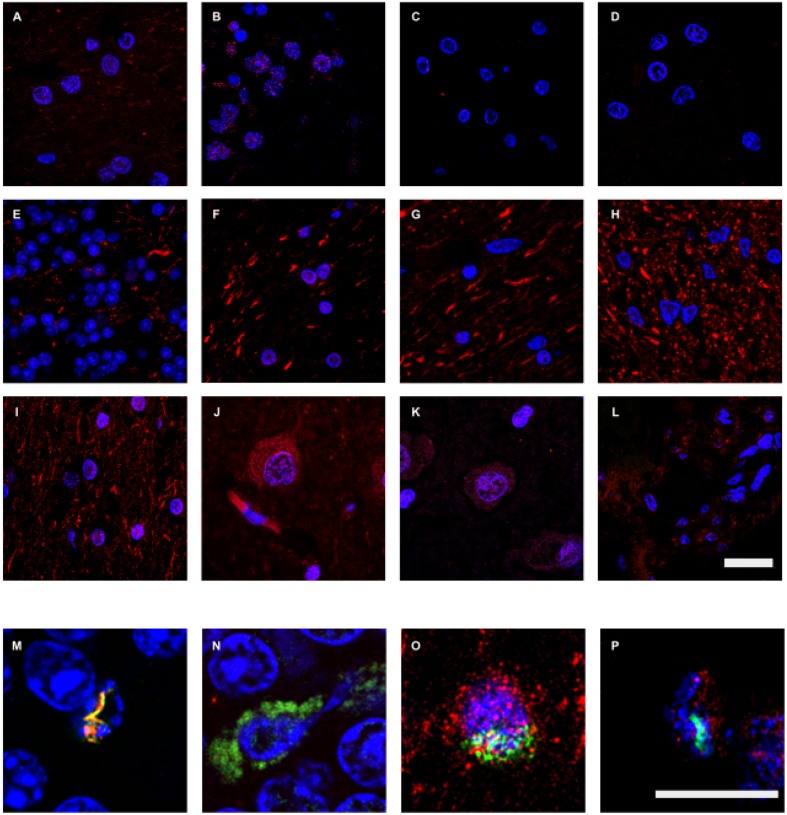
**Immunohistochemistry analysis of fungal enolase, *β*-tubulin, and chitin in brain sections from control subjects.** CNS sections from six control subjects (C1–C6) were analyzed by confocal microscopy as detailed in the section of “MATERIALS AND METHODS.” Sections were immunostained with rabbit polyclonal antibodies against enolase, β-tubulin, and chitin (green) and human α-tubulin and neurofilaments (red), as described in **Figures 1** and **2**. **(A–D,M)**: anti-enolase and anti-human α-tubulin antibodies. **(E–H,N)**: fungal anti-β-tubulin and human anti-neurofilaments antibodies. **(I–L,O–P)**: anti-chitin and anti-human α-tubulin antibodies. **(A,I,O)**: C1. **(B,E)**: C2. **(C,F)**: C3. **(D,N)**: C4. **(G–H,J,M)**: C5. **(K–L,P)**: C6. **(A,G,I,J,O)**: LFC. **(B,E,H,M,N)**: CEC. **(C,D,F,K,P)**: ERH. **(L)**: CP. DAPI staining appears in blue. The different panels are indicated in the figure. Scale bar: 20 μm for panels **(A–L)** and 10 μm for panels **(M–P)**.

Previous work from our group has found that control subjects do not contain appreciable amounts of fungal proteins in brain sections (Pisa et al., 2015a,b). Nevertheless, to compare the burden of chitin, enolase and β-tubulin with AD brain tissue, we performed immunohistochemistry on brain sections from control individuals. We evaluated six control subjects (described in **Supplementary Table S1**) and the more representative results are shown in **Figure 6**. We failed to detect chitin immunoreactivity in brain tissue from control subjects (**Figure 6I–L**). Similar results were obtained using anti-enolase and anti-β-tubulin (**Figure 6A–H**). Rare positivity could be detected in control samples (**Figure 6M–P**). Nevertheless, this low burden of fungal infection in control subjects may be of interest to understand some symptoms such as the stimulation of the immune system in elderly people (Cribbs et al., 2012; Heneka et al., 2015). In conclusion, immunohistochemistry analyses with three new antibodies comprehensively demonstrate the presence of fungal components in brain tissue from AD patients, but these fungal structures are very scanty in control individuals.

## Discussion

Increasing evidence points to the possibility that the etiology of AD is microbial infection (Harris and Harris, 2015). In this regard, we have provided compelling evidence that supports the notion that mixed fungal infections occur in AD. Indeed, several fungal components can be found in brain tissue from AD patients (Alonso et al., 2014a,b, 2015b; Pisa et al., 2015a,b), and fungal proteins and polysaccharides in blood serum, consistent with the concept of disseminated fungal infection (Alonso et al., 2014a). Fungal DNA and proteins are also detected in CSF from AD patients (Alonso et al., 2015b), and proteomic analysis of brain tissue from AD patients unequivocally reveals the presence of fungal proteins and DNA from a variety of fungal species (Alonso et al., 2014b; Pisa et al., 2015b). Collectively, these studies show that AD patients have mixed fungal infections that can be detected directly in brain sections and also in blood serum and CSF.

Our present findings indicate that fungal components such as chitin, enolase and β-tubulin, can be detected with specific antibodies. Several former observations can be reconciled with our present results. Thus, the existence of chitin-like material in AD brains stained with calcofluor (Castellani et al., 2005, 2007) can be easily explained if we consider that our present observations provide strong evidence for fungal chitin in AD brains, which is in accord with the finding of elevated inducible chitinase in serum and CSF from some neurodegenerative diseases, including AD (Choi et al., 2011; Rosen et al., 2014). This is consistent with the existence of disseminated fungal infections since the presence of the substrate (chitin) would induce synthesis of the enzyme (Theis and Stahl, 2004). In fact, elevated levels of chitinase may constitute a good biomarker for early diagnosis and to monitor the evolution of AD (Watabe-Rudolph et al., 2012; Wildsmith et al., 2014).

The amyloid-cascade hypothesis posits that the unregulated synthesis of Aβ and the concomitant formation of extracellular amyloid plaques leads to intracellular neuron injury that is mediated by the formation of tangles of phosphorylated tau protein (O’Brien and Wong, 2011; Revett et al., 2013; Hampel et al., 2015). The identification of Aβ as a potent anti-bacterial and anti-fungal peptide (Soscia et al., 2010), bolsters the claim that microbial infection of AD patients may be responsible for the induction of Aβ synthesis. Indeed, a recent study in experimental animals showed that Aβ is involved in combating bacterial and fungal infections (Kumar et al., 2016). In this sense, Aβ can be envisaged as part of the innate immune response to microbial infections. Kumar et al. (2016) have suggested that a putative infection could trigger the synthesis of Aβ leading to amyloid deposition and neuronal death. In this regard, the main cause of neuronal damage could still be ascribed to amyloid plaques and the inhibition of their formation may alleviate clinical symptoms. Another view, and one that we favor, is that the existence of a fungal infection *per se* is responsible for cell damage and for the majority of clinical symptoms observed in AD (Alonso et al., 2014b; Pisa et al., 2015b). Many fungal species can synthesize toxins that could be detrimental for cellular metabolism (Kamei and Watanabe, 2005; Russo et al., 2016). For example, very recently a new fungal toxin, candidalysin, has been identified in *C. albicans* (Moyes et al., 2016), which targets cellular membranes, leading to increased membrane permeability and lysis. Even for fungal species that do not synthesize mycotoxins, the continuous secretion of lytic enzymes could contribute to their virulence and pathogenesis for cells. Indeed, a number of lytic enzymes that digest proteins and lipids are secreted by fungi (Braga-Silva and Santos, 2011). This would be particularly detrimental for neurons containing intracellular fungi, as described here and in other studies (Pisa et al., 2015a,b). It must also be considered that the continuous secretion of polyglucans will have deleterious effects on cell metabolism and on the immune system (Drummond and Brown, 2011; Saijo and Iwakura, 2011; van der Meer et al., 2015). Therefore, it would be possible that the inhibition and eradication of fungal infection from brain tissue would be sufficient not only to ameliorate, but perhaps to partially revert AD clinical symptoms.

To test if AD symptoms are reversible, clinical trials are needed to evaluate different anti-fungal agents for the evolution of AD symptoms. Indeed, at least two patients diagnosed with AD and treated with anti-fungal compounds exhibited a good recovery from dementia (Ala et al., 2004; Hoffmann et al., 2009). In these two cases, amyloid deposition was not targeted, but a good recovery was still observed. However, it was concluded that these patients were possibly misdiagnosed and in fact could have had cryptococcal meningitis. Nevertheless, if AD is provoked by fungal infections, the test for fungal meningitis would be positive in most cases. Therefore, if dementia provoked by a fungal infection can be reversed by anti-fungal treatment (Ala et al., 2004; Hoffmann et al., 2009), a similar situation could be envisaged if AD dementia is the consequence of mycoses.

Aβ, as well as other small peptides, such as defensins, form part of the innate immune response and are elevated in AD patients (Williams et al., 2013; Watt et al., 2015; Mattar et al., 2016). The use of transgenic mice that express Aβ or defensins have provided evidence that these small peptides play an important role in the innate immune response against microbial infections (Salzman et al., 2003; Chu et al., 2012; Kumar et al., 2016). Defensin-1 is apparent in astrocytes of the AD hippocampus and choroidal plexus (Williams et al., 2013). Moreover, elevated levels of α-defensins-1 and 2 are found in serum from peripheral blood of AD patients (Watt et al., 2015). These observations indicate an inflammatory reaction in these patients, which is consistent with our suggestion of mixed disseminated mycosis.

## Author Contributions

DP and RA carried out the experiments. AR managed the human brains and provided the tissue sections. MH provided the chitin antibody. LC designed the experiments and wrote the manuscript.

## Conflict of Interest Statement

The authors declare that the research was conducted in the absence of any commercial or financial relationships that could be construed as a potential conflict of interest.

The reviewer JL and handling Editor declared their shared affiliation, and the handling Editor states that the process nevertheless met the standards of a fair and objective review.

## Supplementary Material

**FIGURE S1 | Characterization of the antibodies employed in this work.** **(A)** The commercial kit Euroimmun was used for immunofluorescence analysis of rabbit polyclonal antibodies against enolase protein at 1:10 dilution, and β-tubulin, and chitin proteins at 1:50 dilution. The donkey anti-rabbit IgG secondary antibody conjugated to Alexa 488 (Invitrogen) was used at 1:500 dilution. Scale bar: 5 μm. **(B)** Western blotting of *C. famata* and HeLa cells proteins with fungal peptide anti-β-tubulin-KLH at 1:200 dilution, fungal enolase at 1:200 dilution and human α-tubulin at 1:500 dilution, as primary antibodies; donkey anti-rabbit IgG horseradish peroxidase at 1:5000 dilution as secondary antibody for β-tubulin, enolase and control; sheep anti-mouse IgG horseradish peroxidase antibody at 1:1000 dilution as secondary antibody for α-tubulin. Control: without primary antibody. **(C)** Immunofluorescence of HeLa cells cultured and incubated with chitin, enolase and β-tubulin antibodies at 1:50 dilution (green), and human α-tubulin at 1:50 dilution (red). DAPI staining appears in blue. Scale bar: 20 μm.

**FIGURE S2 | Sections (5 μm) of the entorhinal cortex of patient AD5 were double immunostained with anti-enolase (green) and anti-α-tubulin (red) antibodies (Left panel).** A control without anti-rabbit polyclonal antibodies was carried out (Middle panel) or without secondary antibodies against mouse monoclonal antibodies (Right panel). DAPI staining is in blue. Scale bar: 30 μm.

**FIGURE S3 | Immunohistochemistry of AD brain sections.** Sections (5 μm) of the entorhinal cortex of patients AD1 and AD2 were double immunostained with anti-chitin (green) and anti-β-tubulin (red) antibodies. Scale bar: 10 μm.

**TABLE S1 | Age and gender of subjects analyzed in this study.**
