# Supplementary figures and images for: Fungal Enolase, β-Tubulin, and Chitin Are Detected in Brain Tissue from Alzheimer’s Disease Patients
Source: Front Microbiol. 2016 Nov 7;7:1772. doi: 10.3389/fmicb.2016.01772 (PMC5097921; doi:10.3389/fmicb.2016.01772)

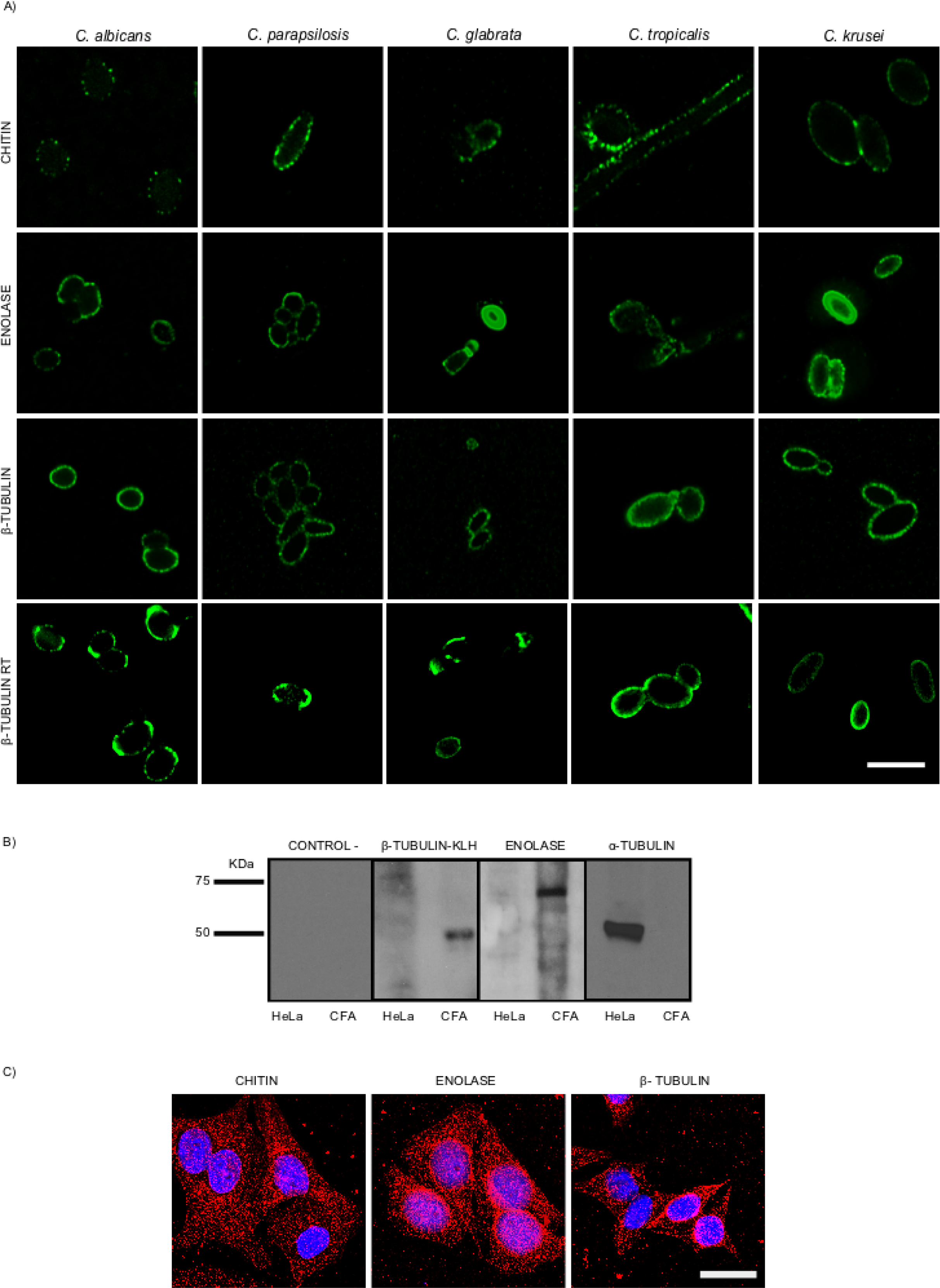

Supplement: Supplementary file 1 [file Image_1.TIF]

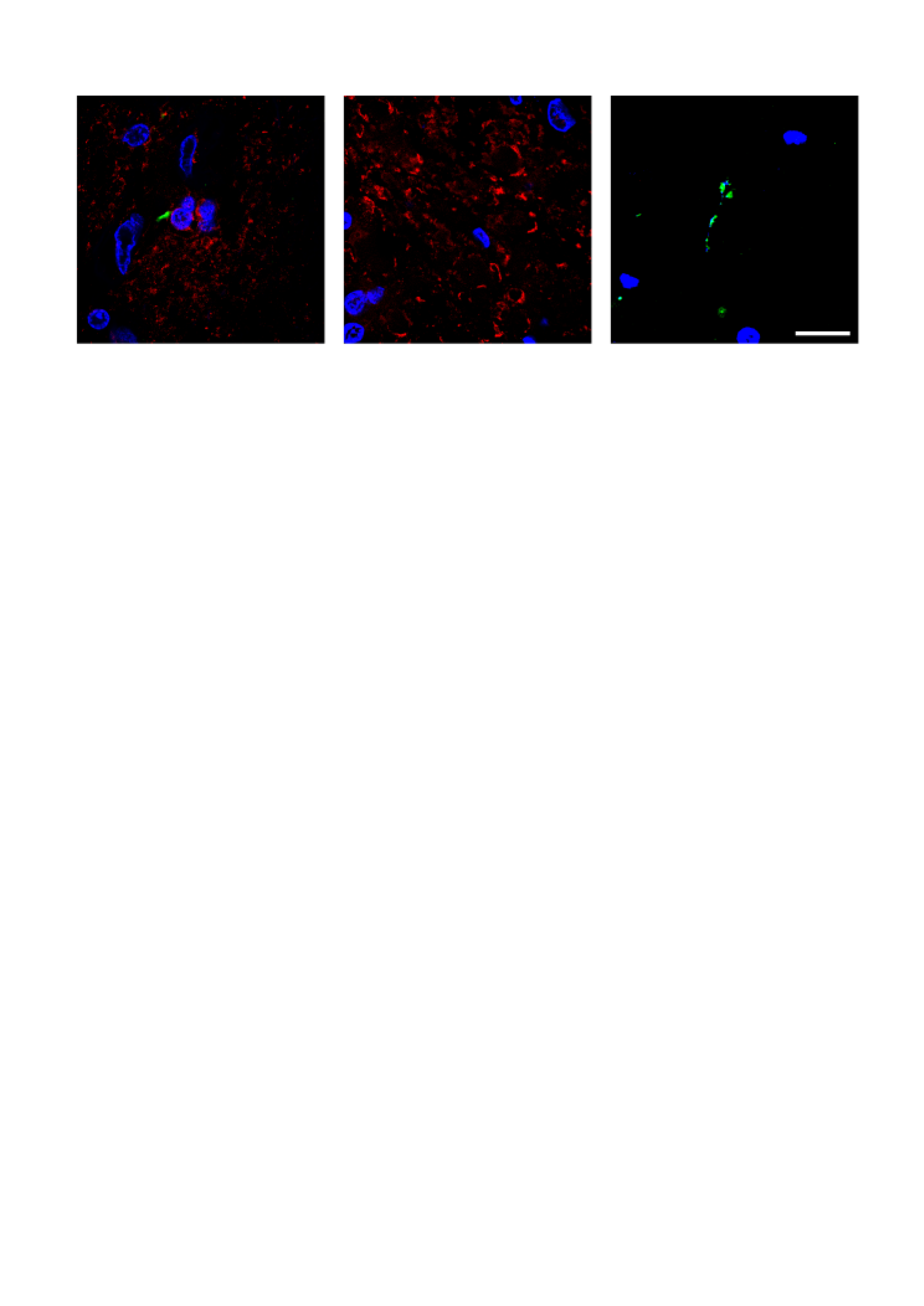

Supplement: Supplementary file 2 [file Image_2.TIF]

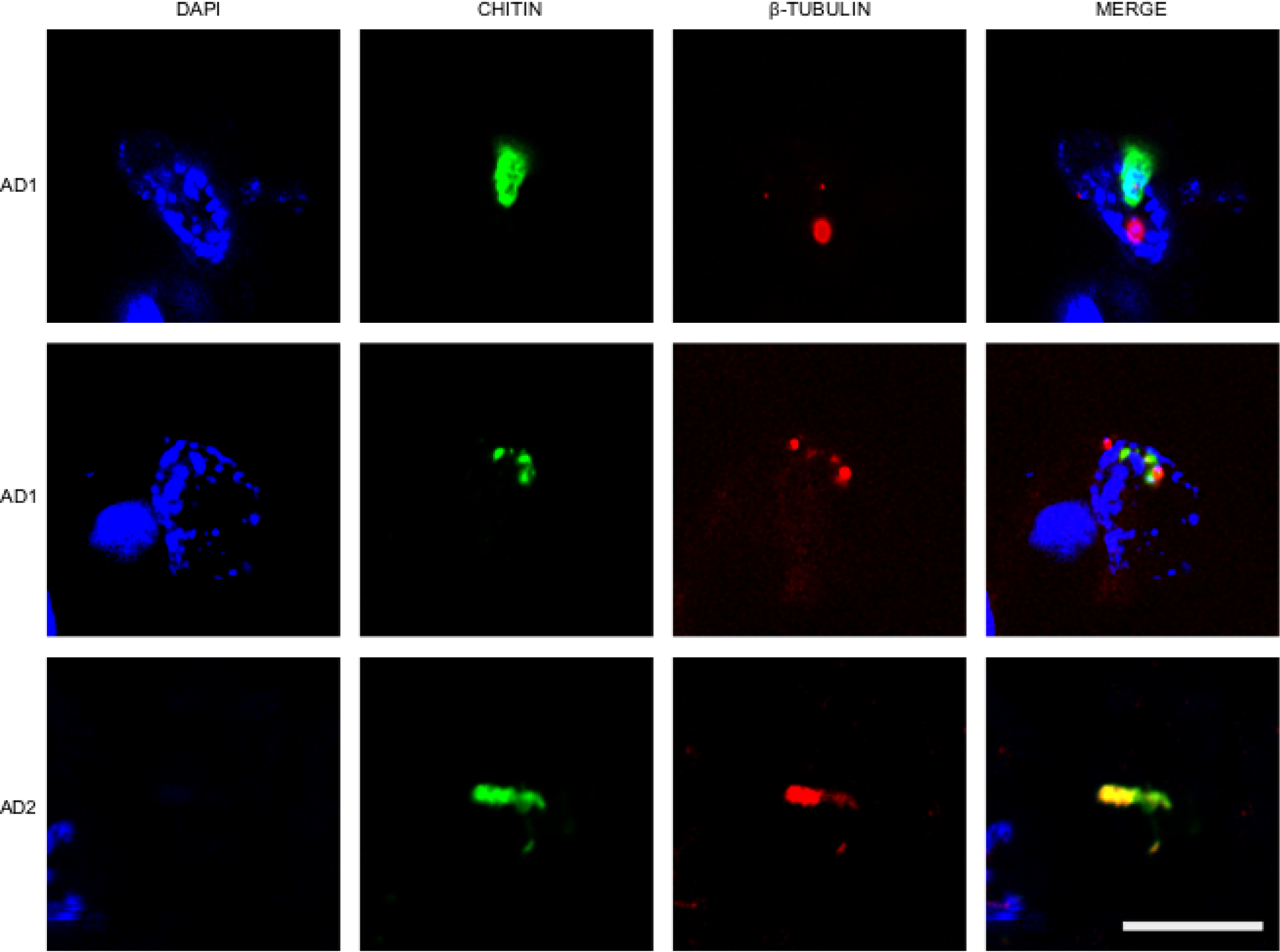

Supplement: Supplementary file 3 [file Image_3.TIF]
