# Supplementary material for: Fungal Enolase, β-Tubulin, and Chitin Are Detected in Brain Tissue from Alzheimer’s Disease Patients
Source: Front Microbiol. 2016 Nov 7;7:1772. doi: 10.3389/fmicb.2016.01772 (PMC5097921; doi:10.3389/fmicb.2016.01772)
Supplement: Supplementary file 4 [file Table_1.DOCX]

| **SAMPLE** | **GENDER** | **AGE** |
| --- | --- | --- |
| AD1 | FEMALE | 80 |
| AD2 | FEMALE | 84 |
| AD3 | FEMALE | 79 |
| AD4 | FEMALE | 81 |
| AD5 | MALE | 87 |
| AD6 | MALE | 92 |
| AD7 | MALE | 81 |
| AD8 | FEMALE | 87 |
| AD9 | FEMALE | 86 |
| AD10 | MALE | 62 |
| AD11 | FEMALE | 83 |
| C1 | MALE | 63 |
| C2 | MALE | 78 |
| C3 | FEMALE | 55 |
| C4 | FEMALE | 62 |
| C5 | MALE | 84 |
| C6 | FEMALE | 77 |

Supplementary table 1
